# Pareidolia in Infants

**DOI:** 10.1371/journal.pone.0118539

**Published:** 2015-02-17

**Authors:** Masaharu Kato, Ryoko Mugitani

**Affiliations:** Human Information Science Laboratory, NTT Communication Science Laboratories, Atsugi, Kanagawa, Japan; Harvard Medical School, UNITED STATES

## Abstract

Faces convey primal information for our social life. This information is so primal that we sometimes find faces in non-face objects. Such illusory perception is called pareidolia. In this study, using infants’ orientation behavior toward a sound source, we demonstrated that infants also perceive pareidolic faces. An image formed by four blobs and an outline was shown to infants with or without pure tones, and the time they spent looking at each blob was compared. Since the mouth is the unique sound source in a face and the literature has shown that infants older than 6 months already have sound-mouth association, increased looking time towards the bottom blob (pareidolic mouth area) during sound presentation indicated that they illusorily perceive a face in the image. Infants aged 10 and 12 months looked longer at the bottom blob under the upright-image condition, whereas no differences in looking time were observed for any blob under the inverted-image condition. However, 8-month-olds did not show any difference in looking time under both the upright and inverted conditions, suggesting that the perception of pareidolic faces, through sound association, comes to develop at around 8 to 10 months after birth.

## Introduction

Faces are important for our social life. We are exposed to lots of faces and have to quickly recognize them in order to deal with social activities. Perhaps because of a side effect of quick face perception, we find faces in inanimate objects such as in the front view of cars and trains and in spots on walls and ceilings. This tendency to project something not actually there onto a different object is known as pareidolia. Once we perceive pareidolic faces, it is hard to ignore them even if we know that they are not real. This pareidolic face perception has been confirmed by behavioral experiments [1–3].

In brain-activity measurements [4,5], the same event-related brainwave component was observed during the presentation of images of real faces and pareidolic faces. This component is known as N170 or M170 and its source is thought to be the fusiform face area (FFA) [5,6] (but see, [7]). Since this cortical area is associated with the center of face identification in the cortex [8,9], observers might not be able to perceive a pareidolic face if the brain in that area has not biologically/functionally matured yet. Thus, in the current study, we attempted to reveal whether and when such pareidolic face perception occurs in infants. For this purpose, we used sound-mouth association in infants.

To demonstrate that infants can find a face in non-face image behaviorally, we have to find a response that is only observable when they recognize a face. One possibility is to use their orientation behavior toward a sound source. Since the mouth is the unique sound source in a face, we can determine the location of the mouth from their looking direction during sound presentation. Indeed, when a visual image of an actor’s face and her monologue were presented simultaneously, 8- to 12-month-olds looked longer at the mouth area than at the eye area [10]. This sound-mouth association was also demonstrated in another study, where 6-month-olds looked longer at the mouth area when the actor on the screen talked to them than when the actor just smiled [11]. Another line of research has shown that infant voice perception is modified by the concurrent mouth shape by 6 months of age at the latest [12–16], suggesting that infants older than that implicitly regard the mouth as the most relevant place for a voice.

Thus, we prepared a very simplified image comprising four blobs with a contour (two blobs for eyes, one lower blob for the mouth, and one top blob as a distractor). Since other information such as color and shape uniquely found in the mouth is not in the image, the presence/position of the mouth is only discerned from the configuration of the blobs. An infant’s looking at the mouth area when sound is heard from this simplified face-image would indicate that the infant regards the image as a face.

**Fig 1:**
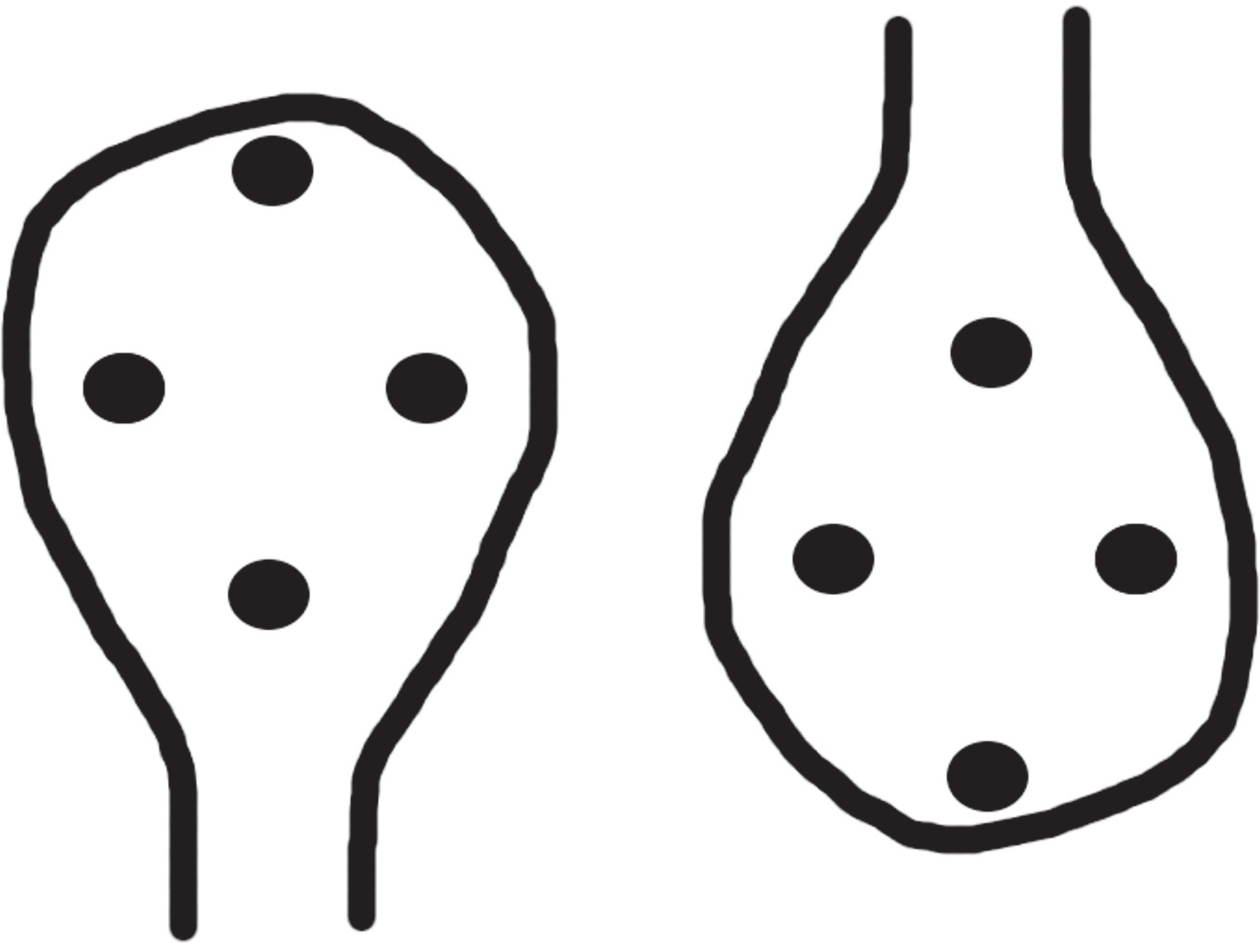
The upright (left) and inverted (left) images presented to infants. Three areas of interest (AOIs) were set to measure the looking time to the blobs. In the upright image, the ‘top’ area covers the upper blob, and the ‘bottom’ area includes the lower blob. The two areas that cover left and right blobs were virtually concatenated as one AOI and the area is called the ‘middle’ area.

We also take care about the sound we presented. We chose a pure tone, rather than the human voice, as an auditory stimulus in order to avoid the possibility that the sound itself would facilitate infants’ perception of a face in the blobs. As another twist in the current experiment, we added a top blob in the image as a distractor. The configuration of the blobs is then diamond-like as shown in Fig. 1. Since our purpose is to explore infant pareidolic face perception, our making the image less face-like suits this objective. It is known that young infants have a preference to look at top-heavy images [17], i.e., images whose components are located more in the upper half of an image (like the eyes) than in the lower half (like the mouth). Removing the top-heavy characteristics from target image makes it possible to interpret the results aside from their automatic top-heavy response.

## Methods

The study was conducted in accordance with the ethical standards specified in the 1964 Declaration of Helsinki and was approved by the ethics committee of Doshisha University, where the experiment was conducted. Prior to each experiment, the families of the participating infants were informed of the study's purpose and signed consent forms. Infants and their parents received a token worth 1,000 Japanese yen as compensation. Sixteen healthy, full-term 8-month-old infants (eight males and eight females, 241 ± 3.8 days old [mean ± standard deviation (SD)]), 16 10-month-old infants (seven males and nine females, 305 ± 10.7 days old), and 14 12-month-olds infants (eight males and six females, 361 ± 6.7 days old) participated in this study.

The infants in our study were shown an image containing one contour and four blobs, equally spaced to form a diamond inside the contour (Fig. 1). The visual stimuli were presented on a 17-inch LCD display. A cornea-reflection eye-tracking system (Tobii X120, Tobii Corp.), which measures the direction of each eye separately at 60 Hz, was attached to the bottom of the display. The sound emanated from two 6-cm full-range loudspeakers located just behind the display. Since the same sound was presented with the same amplitude, the sound image was located at the center of the screen. We confirmed beforehand that it was impossible for adults to determine which blob was likely the sound source solely from the direction of the sound. The distance between the participants and the display was set at approximately 60 cm to facilitate efficient eye tracking. At this distance, the image subtended 14.3° × 21.5°, the distance between the left and right blobs was about 8°, and the distance between the top and bottom blobs was about 10°.

The experiment was conducted in a quiet, dark room. Each infant sat on the lap of a parent. After the lights had been turned off, the calibration procedure for the eye tracker was initiated. We used a five-point calibration procedure. A short expanding-contracting nonface-like object with accompanying sound appeared on the display and when the experimenter was certain that the participant was looking at it, the experimenter pushed a key and proceeded to the next calibration point. The order was randomized. Completing the calibration procedure took 1 min on average. Subsequently, inverted, then upright test images were presented serially for 30 s each. The fixed order of stimuli presentation was used in order to avoid carry-over effect, i.e., once the image was regarded as face in the upright position, the perception could persist in inverted position. Between the test stimuli, a short video clip of small nonface-like objects with accompanying sounds were shown in the middle of the screen as attention-getters. The clip was shown until the participants looked at the center of the screen. To eliminate the effect of the initial fixation point on the time spent looking at each blob, the image was presented at either the left or the right side of the display; that is, the image center was 8° left or right from the screen center, and this was counterbalanced between subjects.

During the presentation of a test image, no sound was presented for the first 15 s. During the remaining time, a pure tone (400 Hz, 51 dB SPL) was intermittently presented with an interval of 1 s. Data for indexing their gaze in response to test stimuli were collected. The gaze points were the averages of both eyes’ raw gaze data, and if the gaze from one eye was lacking, the averaged gaze point was also lacking. We counted the number of times that the averaged gaze points fell within the areas of the whole screen and each of the four blobs and calculated the looking time from the result. For simplicity, we call the areas of interest (AOIs) the ‘middle’ (areas for the left and right blobs were virtually concatenated as one AOI), ‘bottom’ (lower blob area in the upright condition, or upper blob area in the inverted condition), and ‘top’ (upper blob area in the upright condition, or lower blob area in the inverted condition).

## Results

### Effect of sound on the change in looking time ratio

**Fig 2:**
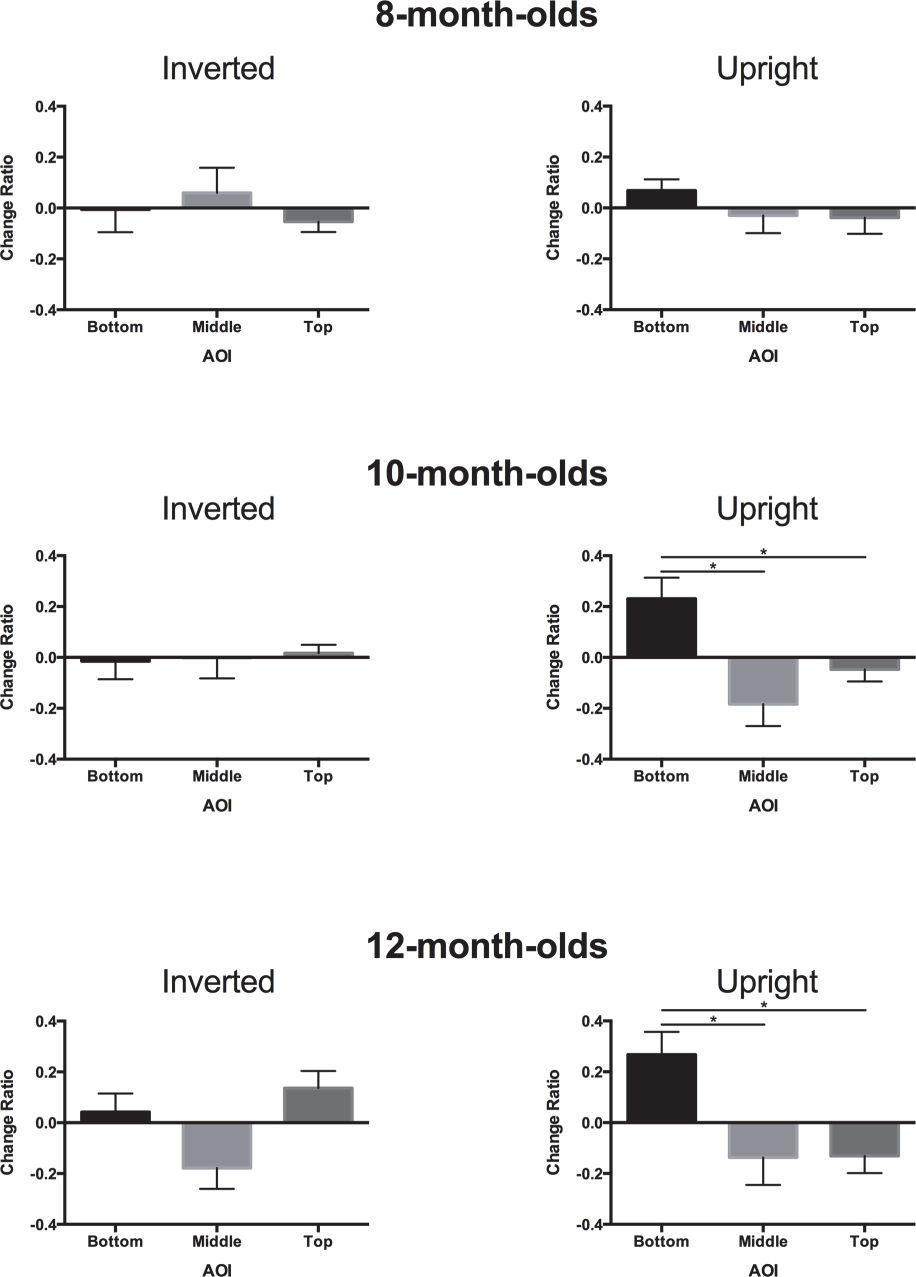
Looking-time change ratios. The left side panels present looking-time change ratios as a function of area of interest (AOI) for the inverted image from the silent to sound conditions. The right side panels present data for the same dependent variable as the left side, but under the upright-image condition. Data obtained from 8-, 10-, and 12-month-old infants are shown in top, middle, and bottom panels, respectively.

Since our primary interest is whether the looking time to the bottom increased after sound presentation, we compared the change in the looking time ratio with and without sound. For the comparison, the looking times for each AOI were normalized by the sum of the looking times for the three AOIs. Then the change ratio for each AOI was defined as the difference in the normalized looking time from the silence condition to the sound condition. A one-way repeated measures analysis of variance on the change ratios for upright images in 10-month-olds showed a different looking time for AOIs, with a larger change ratio for the bottom than for the top and middle(*F*(2,30) = 5.433, *p* = .010, η^2^ = 0.266). This was statistically confirmed by post-hoc multiple comparison, where looking time for the bottom area increased more than that for the middle area (*t*(15) = 2.565, *p* = .034) (adjusted by Shaffer’s modified sequentially rejective Bonferroni method; this method was also applied in post-hoc multiple comparison elsewhere in this study) and more than that of the top area(*t*(15) = 2.674, *p* = .034) (see Fig. 2, middle row). In contrast, the inverted image yielded no significant increases in terms of the change ratio (*F*(2,30) = 0.041, *p* = .960, η^2^ = 0.003).

The same tendency was observed in the 12-month-old infants. The one-way repeated measures ANOVA on change ratios for upright images showed different looking times for each AOI (*F*(2,26) = 4.435, *p* = .022, η^2^ = 0.254). This was statistically confirmed by post-hoc multiple comparison, which showed looking time for the bottom area increased more than for the middle area (*t*(13) = 2.174, *p* = .049) and more than that for the top area (*t*(13) = 3.433, *p* = .009). The inverted image yielded marginally significant differences in change ratios (*F*(2,26) = 3.165, *p* = .059, η^2^ = 0.196)(see Fig. 2, bottom row). Since the significance is marginal, we conducted post-hoc multiple comparison analyses, but the differences between the bottom and other AOIs were not significant (*t*(13) = 1.595, *p* = .135 and *t*(13) = 0.819, *p* = .428, for bottom vs. middle areas and bottom vs. top areas, respectively).

However, eight-month-olds behaved differently. The one-way repeated measures ANOVA on change ratios with AOI as the factor yielded no statistically significant differences for the upright image (*F*(2,30) = 0.652, *p* = .528, η^2^ = 0.042) or for the inverted image (*F*(2,30) = 0.331, *p* = .721, η^2^ = 0.022) (see Fig. 2, top row).

### Looking times as functions of age, orientation of image, and AOI without sound presentation

On average, infants looked at the screen for 8.11s out of 15 s of stimulus presentation. The results were analyzed using a three-way mixed measures ANOVA with infant age, orientation of image, and presence of sound as factors. There was a main effect of presence of sound (*F*(1,43) = 12.958, *p* = .001, η^2^ = 0.027). Since the sound condition always followed the silent condition, infants looked at the screen longer in the silent condition [8.65 ± 3.54 s (mean ± SD)] than in the sound condition(7.57 ± 2.94 s). There were no other main effects and no two-way or three-way interactions.

Next, we investigated which part of the stimuli drew infants’ attention in a natural situation without sound presentation. As preparation, we normalized the looking time to each AOI on the basis of its size. We did this because if we assume the gaze to be equally distributed across the blobs, we may expect the middle area including two blobs to be gazed at for a longer time, compared to the top and bottom areas with only one blob each. A three-way mixed measures ANOVA on looking time in the silent condition with age, AOI, and orientation of image as factors revealed an interaction effect of AOI and orientation (*F*(2,86) = 5.874, *p* = .004, η^2^ = 0.043). Subsequent post-hoc analyses showed that the looking time was significantly different among AOIs for the inverted image (*F*(2,86) = 12.004, *p*< .0001, η^2^ = 0.157), whereas the difference was not observed for the upright image(*F*(2,86) = 0.693, *p* = .503, η^2^ = 0.011). When inverted, they looked longer at the middle area (1.36±0.84 s)and bottom area (1.46±1.46 s) than at the top area (0.45±0.72 s) (*t*(43) = 5.388, *p*< .0001 for between middle and top areas;*t*(43) = 3.638, *p* = .001, for between bottom and top areas). Other two-way and three-way interaction effects were not significant. The main effect of AOI was significant (F(2,86) = 7.083, p = .001, η^2^ = 0.047); they looked longer at the middle area (1.22±0.87s)than at the top area (0.68± 0.99 s, *t*(43) = 3.459, *p* = .004), and looked longer at the bottom area (1.14±1.31 s) than the top area (*t*(43) = 2.809, *p* = .008), but no difference was found between the middle and bottom(*t*(43) = 0.604, *p* = .549).

Since there were two blobs in the middle AOI, the bias of looking at either the left or right blob in that AOI was examined. A three-way mixed measures ANOVA in the silent condition with age, presence of sound, and orientation as factors found no main or interaction effects, suggesting no preference towards the left or right blob in the middle AOI. However, infants showed left/right bias in terms of the relative position of the image on the screen. They looked at the middle blob that was close to the center of the screen (1.94 ± 1.70 s)longer than at the one that was close to the edge of the screen, (0.46 ± 0.80 s, *t*(91) = 7.807, *p* < .0001). This suggests that the duration of the looking time was dependent on the distance from the screen center, which is where they initially looked because the screen center was the attention getter.

## Discussion

The finding that infants looked longer at the bottom blob in the upright image permits the inference that infants are sensitive to a relationship between the sound and the bottom blob. Because the pure tone did not resemble a human voice, it is difficult to assume that this tone directly helped infants to perceive the image as a face. Rather, it is more reasonable to assume that infants "knew" in advance about the existence of the mouth through the configuration of blobs and contour and thus looked at the bottom blob as the most relevant area for this sound. Assuming that this interpretation is correct, we may infer that the bottom blob would not be regarded as the mouth when the image is difficult to perceive as a face; hence, we would not expect this blob to be viewed longer under the sound condition than under the silent condition. Data for the inverted image confirm this inference.

Several studies have investigated brain activity during multisensory perception. A study that used near-infrared spectroscopy to examine cortical brain activity to audio-visual stimuli in 3-month-olds suggested that not only modality-specific brain areas but also the association areas of the temporal and parietal cortices and the anterior prefrontal regions are already able to respond to multisensory stimuli [18]. Using the McGurk effect [19], an illusory speech percept by concurrent visual speech cues, an electroencephalography study suggested that temporal and frontal brain areas are able to respond to this multisensory illusion [20]. Taken together, these findings indicate that infants in the current study were able to process our stimulus as a multisensory one.

The infants in the current study looked at the three AOIs in the upright image equally in the silent condition. In contrast, the eyes were the areas most looked in a face in previous studies (e.g., [21–24]). That difference might be because of the stimuli used in experiments. In previous studies, real faces or photographic/schematic images of faces have been used (e.g., [21–24]), whereas a pareidolic face was used in the current study, and as far as we know, no study except the current one has measured the looking time to face parts in pareidolic face. A pareidolic face differs from a photographic/schematic face in terms of decreased information relevant to a face. In addition, our pareidolic face contains a distractor (top blob) that would draw the observer’s attention. Therefore, these factors would decrease the looking time towards the eye area in our pareidolic face.

Since the distance between the sound image (around the center of the screen) and the position of the four blobs were a bit different, one may suspect that the gradual development of sound localization ability could explain the result. However, we do not think this is plausible, because the relative position of the sound image and four blobs were the same in the upright and inverted conditions, which cannot explain why the increase in looking time to the bottom blob was observed in only the upright condition.

The current results indicate that infants 8 to 10 months of age come to experience pareidolia. Since the FFA plays a primal role for pareidolia [5], our result suggests that it is functional at least at that age. However, one study relevant to pareidolic face perception in infants[25] has reported that as early as 7 to 8 months infants can perceive Arcimboldo’s paintings as faces. In Arcimboldo’s paintings, an individual face is created by the aggregation of objects such as vegetables. Although the details of those objects are not the same as those of parts of a face, their shape and color are roughly similar to them. In that sense, Arcimboldo paintings contain a lot of facial information in the low-frequency region, but that information is obscured by the detail (high-frequency region) of the objects that constitute the painting. Compared to Arcimboldo’s paintings, our image is very simplified in that it contains no featural information. The richness of the facial information contained in Arcimboldo’s paintings possibly accounts for the time lag of face detection between them and the four-blob image in our study.

That difference between Arcimboldo’s paintings and our simplified schematic image, together with the pareidolic face perception in adults, even gives us an implication as to what is necessary for the development of pareidolic perception. Our results suggest that only a first-order configural cue is necessary in order for 10-month-olds to perceive a pareidolic face and that additional facial information in an Arcimboldo’s painting helps infants to perceive the painting as a face at 7 to 8 months. In contrast, such rich information is not necessarily required for adults to be able to perceive pareidolic faces. A study has shown that through the multiple regression analysis of adult face perception using face-like objects, the existence of eyes is a significant predictor of variance for pareidolic face perception [3]. Another has even showed that adults can perceive a pareidolic face from a pure noise image and that their right fusiform face area is activated by the stimulus [26]. This might indicate that the role of top-down processing in face perception becomes evident with age.

It is known that infants early in life also prefer to look at face-like objects, but this preference disappears after a half year of life [27]. Accordingly, Johnson et al. [27] argued that the preference for face-like objects is processed in the subcortical area and that the cortical area gradually takes over. Since the current study showed that pareidolic perception comes about during the latter half of the first year of life, the pareidolia we observed would be different from what is observed in neonates.

A pareidolic face is an illusion, but the existence of this illusion indicates the specialty of face processing in humans. Our findings suggest that even 10-month-olds can perceive pareidolia, which is a result of biological/functional maturation of the brain region in the cortex.

## Supporting Information

S1 Data Individual data of change of looking time ratio, looking time to the screen with and without sound presentation, normalized looking time in each AOI without sound presentation, and looking time to eyes. (XLSX)
